# Supplementary material for: Identification of Putative Neuropeptides That Alter the Behaviour of Schistosoma mansoni Cercariae
Source: Biology (Basel). 2022 Sep 12;11(9):1344. doi: 10.3390/biology11091344 (PMC9495596; doi:10.3390/biology11091344)
Supplement: Supplementary file 1 [file biology-11-01344-s001.zip › File S1.pdf]

Identification of Putative Neuropeptides that Alter the Behaviour of *Schistosoma mansoni* Cercariae

Conor E. Fogarty et al.

File S1. Comparative sequence analysis of all precursor proteins identified in this study.

Alignment of Cercariae neuropeptide

Method

Annotation of putative *S. mansoni* neuropeptides was performed by BLASTp search against non-redundant protein database, NCBI (May, 21st 2020). Homologous proteins match with *S. mansoni* neuropeptides with *E*-value≤0.5 were retrieved from NCBI and used for multiple sequence alignment. Amino acid alignment was generated using MEGA X software (version 10.1.8) (Kumar, Stecher et al. 2018) with parameter set as follows: algorithm, ClustalW; gap opening penalty, 10; gap extension penalty, 0.2. Visualization of alignment was carried out on TeXworks software. Sequence logo was generated by using WebLogo application (Crooks et al., 2004).

Table 1. Species and species abbreviations used in the alignments.

Species	Species abbreviation
Clonorchis sinensis	*C. sinensis*
Dugesia japonica	*D. japonica*
Echinococcus granulosus	*E. granulosus*
Echinococcus multilocularis	*E. multilocularis*
Echinostoma caproni	*E. caproni*
Fasciola gigantica	*F. gigantica*
Fasciola hepatica	*F. hepatica*
Fasciolopsis buski	*F. buski*
Macrostomum lignano	*M. lignano*
Mesocestoides corti	*M. corti*
Opisthorchis felineus	*O. felineus*
Opisthorchis viverrini	*O. viverrini*
Paragonimus westermani	*P. westermani*
Schistosoma bovis	*S. bovis*
Schistosoma curassoni	*S. curassoni*
Schistosoma haematobium	*S. haematobium*
Schistosoma japonicum	*S. japonicum*
Schistosoma mansoni	*S. mansoni*
Schistosoma margrebowiei	*S. margrebowiei*
Schistosoma mattheei	*S. mattheei*
Schmidtea mediterranea	*Sch. mediterranea*
Trichobilharzia regenti	*T. regenti*

Npp-1

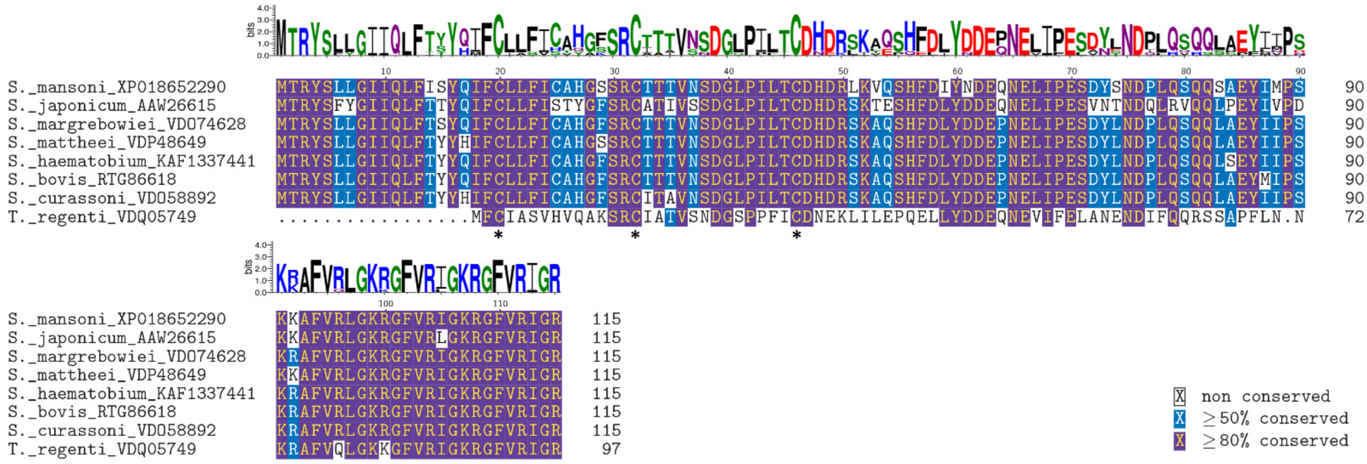

Npp-5

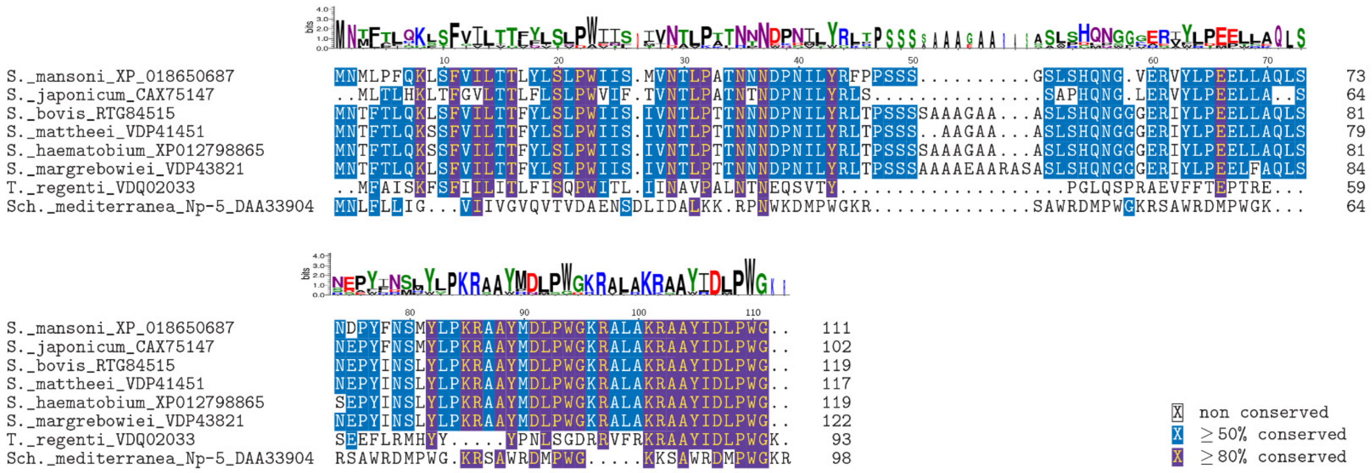

Npp-14

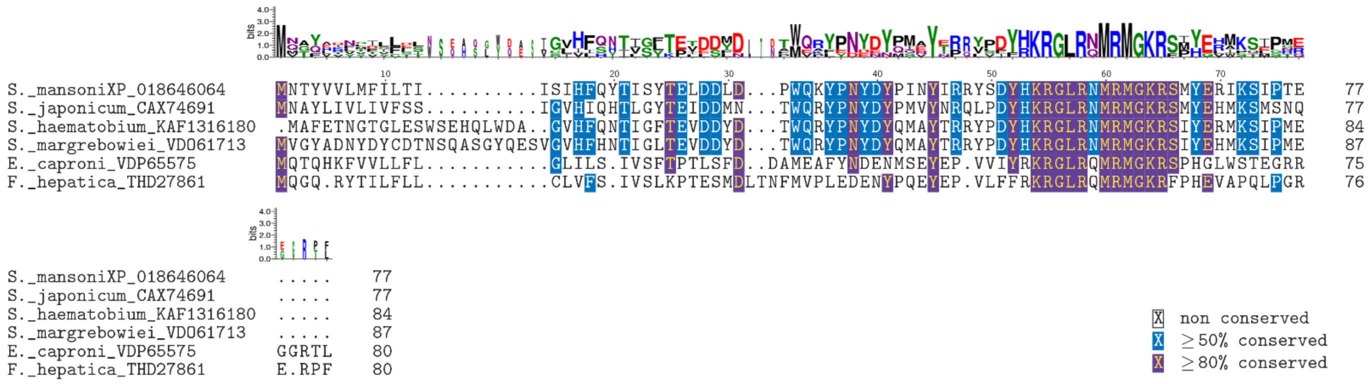

Npp-17

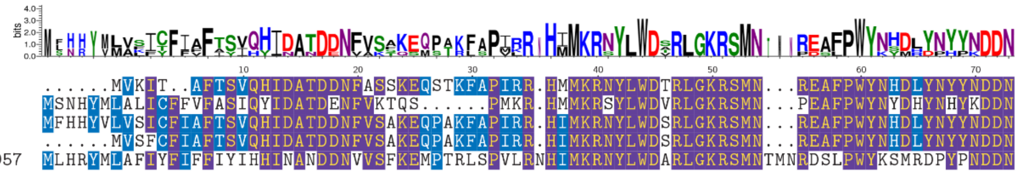

```
S._mansoni_XP018654248              ......MVKIT..AFTSVQHIDATDDNFASSKEQSTKFAPIRR.HMMKRNYLWDTRLGKRSMN...REAFPWYNHDLYNYYNDDN   73
S._japonicum_TNN07359               MSNHYMLALICFFVFASIQYIDATDENFVKTQS......PMKR.HMMKRSYLWDVRLGKRSMN...PEAFPWYNYDHYNHYKDDN   75
S._haematobium_XP_012797838         MFHHYVLVSICFIAFTSVQHIDATDDNFVSAKEQPAKFAPIRR.HIMKRNYLWDSRLGKRSMN...REAFPWYNHDLYNYYNDDN   81
S._curassoni_VDO81289               ......MVSFCFIAFTSVQHIDATDDNFVSAKEQPAKFAPIRR.HIMKRNYLWDSRLGKRSMN...REAFPWYNHDLYNYYNDDN   75
Trichobilharzia_regenti_VDQ03957    MLHRYMLAFIYFIFFIYIHHINANDDNVVSFKEMPTRLSPVLRNHIMKRNYLWDARLGKRSMNTMNRDSLPWYKSMRDPYPNDDN   85
```

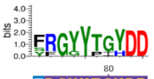

```
S._mansoni_XP018654248              FRGYYTGYDD    83
S._japonicum_TNN07359               SRGYYTIYDD    85
S._haematobium_XP_012797838         FRGYYTGYDD    91
S._curassoni_VDO81289               FRGYYTGYDD    85
Trichobilharzia_regenti_VDQ03957    YFGGYPGHDD    95
```

X non conserved
X ≥ 50% conserved
X ≥ 80% conserved

Npp-26

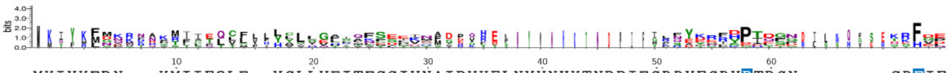

```
S._mansoni_XP018648926                  MKIYKFDN...KMIIEQLF..YCLLVFITFSSIVNAIPVHELNYVNHKTNDPIFGRRYEGRYPTDGN........SRFIE   67
S._margrebowiei_VDP46653                MKIYKLDNNNNKMIIEQLF..YCLIVLIAFSSIIN....................AQYDERYPTDGN........TRFVE   50
S._haematobium_XP012801475              ..............................MIG.....................KQYDERYPTDGN........TRFVE   20
S._japonicum_AAP06214                   MKTYKLNEK..KMFIEQCF..YLLLGLLIFSSTVNAVPVRE.............PNYEN.HGILGNRLGKHFERQYFVE   61
Trichobilharzia_regenti_VDQ00204        .....MNPRHQSTFAQQTMPHHAGQGHYVLSELEE....................NYPRIHETETS...........GN   43
Clonorchis_sinensis_GAA38410            .....MMQKNAARICTLCLVLLYLVEASQNMEEKSMDEQHE.............MFSAYPLSPHESPDILRQVSEDEHEL   62
Opisthorchis_felineus_TGZ67796          .....MMQRKATRIGTLCLVILYLVEASQNEEEKTMDQQRE.............ILSEYSPSPEDSPDILSQGSEYGHGL   62
Macrostomum_lignano_PAA56213            ......MASRNFSTSPLVLLLLLLLGCTAFSAESR.....................TDIKDASSD...........GD   40
Dugesia_japonica-Np23_BAV14853          .....FGK...................KFDPIMFG....................KRFDPIQFG........KKFDP   25
Dugesia_japonica-Np51_BAV14862          ....MFGKRFDP............SQFRMKFDPKMFGDKFDS............YRFEKRFDPIQFG........KKFDP   44
Schmidtea_mediterranea-Npp15_DAA33902   .MQFRFSKMTHVTLLITVGLFHVISGYPQTYELNQSDRGYYP............ILFDKRFDPIQFG........KRFDP   59
Mesocestoides_corti_VDD74267            ......................MVRGPVFVCLSVA.........................HPTTDE...........ED   21
```

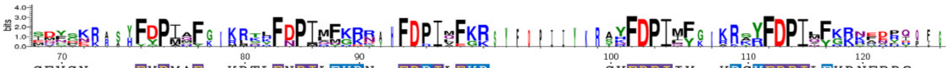

```
S._mansoni_XP018648926                  SEYSN....FYPMAF..KRTLFNPILFKRN..FDPILFKR..........SYFDPIIY..KRSYFDPILFKRNEDRQ...   124
S._margrebowiei_VDP46653                SDYSN....YYPMAL..KRTLFNPILMKRN..FDPIVFKRSYFDPIIYKRSYFDPIIY..KRSYFDPILFKRNENRQ...   117
S._haematobium_XP012801475              SDYSN....FYPMAF..KRTLFNPILMKRN..FDPIVFKRSYFDPIIYKRSYFDPIIY..KRSYFDPILFKRNENRQ...    87
S._japonicum_AAP06214                   SDYPDKRSYFDPIAF..KRTYFDPIAFKRN..FDRILFKR...........NFDPILF..KRSYFDPIAFKRNADHQ...   121
Trichobilharzia_regenti_VDQ00204        ERNPE....FGPAGF..KRN.FDPIMFKRSY.FDPIMFKR..........TYFDPIMF..KRTYFDPIMYKRSSEYH...   100
Clonorchis_sinensis_GAA38410            MDSDKRASHFDPIMFK.RLSHFDPIMFKRRAHFDPIMFKR.........RAHFDPIMFK.RRAHFDPIMFKRRAHFDPI.   130
Opisthorchis_felineus_TGZ67796          MNGDKRASHFDPIMFK.RLSHFDPIMFKRRAHFDPIMFKR.........RAHFDPIMFK.RRAYFDPIMF..........   121
Macrostomum_lignano_PAA56213            EVNAEKRAYFDPINYGKKRAYFDPINYGKK.........R..........AYFDPINYGKKRAYFDPINYGKKRAYY...    98
Dugesia_japonica-Np23_BAV14853          IMFGKR...FDPIQFG...KKFDPIMFGKR..FDPIQFGK...........KFDPIMFG...KRFDPIQFGKKFEP....    79
Dugesia_japonica-Np51_BAV14862          IMFGKR...FDPIQFG...KKFDPIMFGKR..FDPIQFGK...........KFDPIMFG...KRFDPIQFGKKFDPIQFG   102
Schmidtea_mediterranea-Npp15_DAA33902   IQFGKR...FDPIQFG...KRFDPIQFGKR..FDPIQFGK...........RFDPIQFG...KRFDPIQFGKRFDPIQFG   117
Mesocestoides_corti_VDD74267            LDLSKR..YFDPIRF..MMR...PMRNRDD......AEKR..........AYFDPILF..AKRYFDPIIYRHPM......    70
```

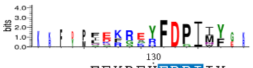

```
S._mansoni_XP018648926                  .....FEKREHFDPIIY..   136
S._margrebowiei_VDP46653                .....LEKREYFDPIIY..   129
S._haematobium_XP012801475              .....LEKREYFDPIIY..    99
S._japonicum_AAP06214                   .....FDKREYFDPIIY..   133
Trichobilharzia_regenti_VDQ00204        .....SD............   102
Clonorchis_sinensis_GAA38410            ....MFKRRAYFDPIMF..   143
Opisthorchis_felineus_TGZ67796          ...................   121
Macrostomum_lignano_PAA56213            .....QSRGGYFDPINYE.   111
Dugesia_japonica-Np23_BAV14853          ............DSIW...    83
Dugesia_japonica-Np51_BAV14862          KRFYPIEFSRRFDPIMFGK   121
Schmidtea_mediterranea-Npp15_DAA33902   KKFDPIQFGKRFDPIMFGR   136
Mesocestoides_corti_VDD74267            ...................    70
```

X non conserved
X ≥ 50% conserved
X ≥ 80% conserved

Npp-30

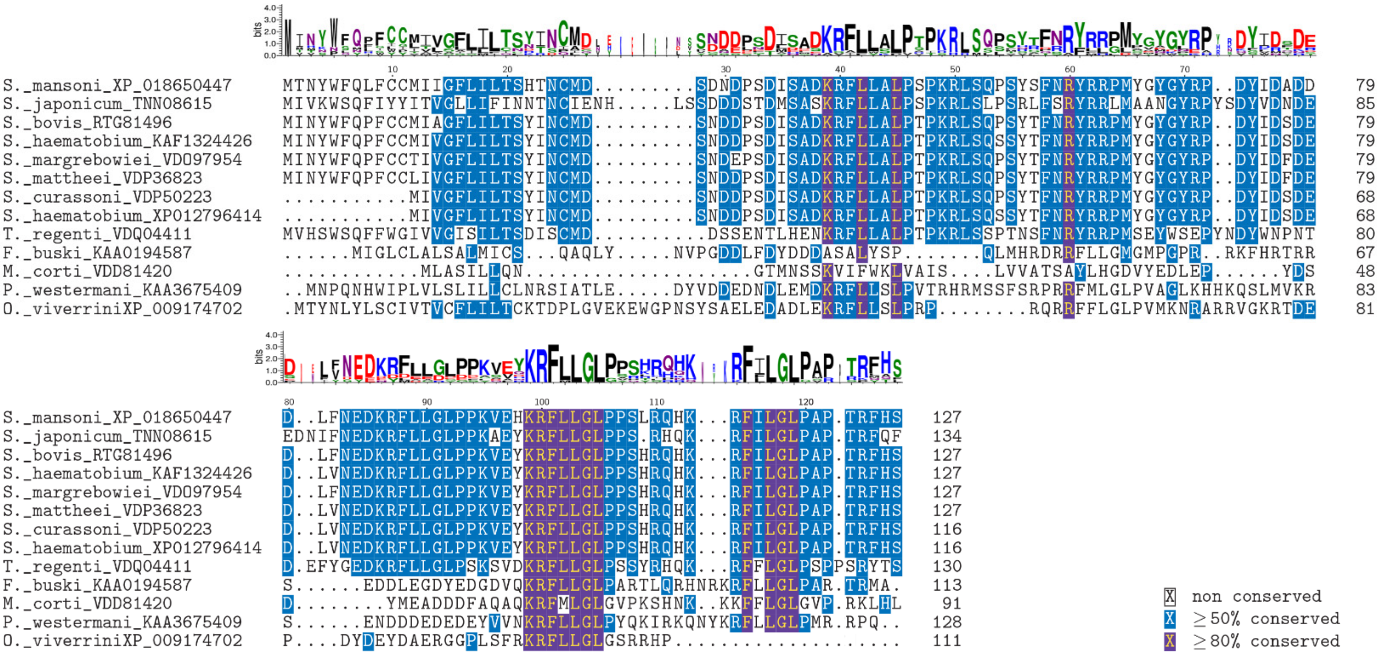
S._mansoni_XP_018650447
S._japonicum_TNN08615
S._bovis_RTG81496
S._haematobium_KAF1324426
S._margrebowiei_VDO97954
S._mattheei_VDP36823
S._curassoni_VDP50223
S._haematobium_XP012796414
T._regenti_VDQ04411
F._buski_KAA0194587
M._corti_VDD81420
P._westermani_KAA3675409
O._viverriniXP_009174702
non conserved
≥50% conserved
≥80% conserved

Npp-31

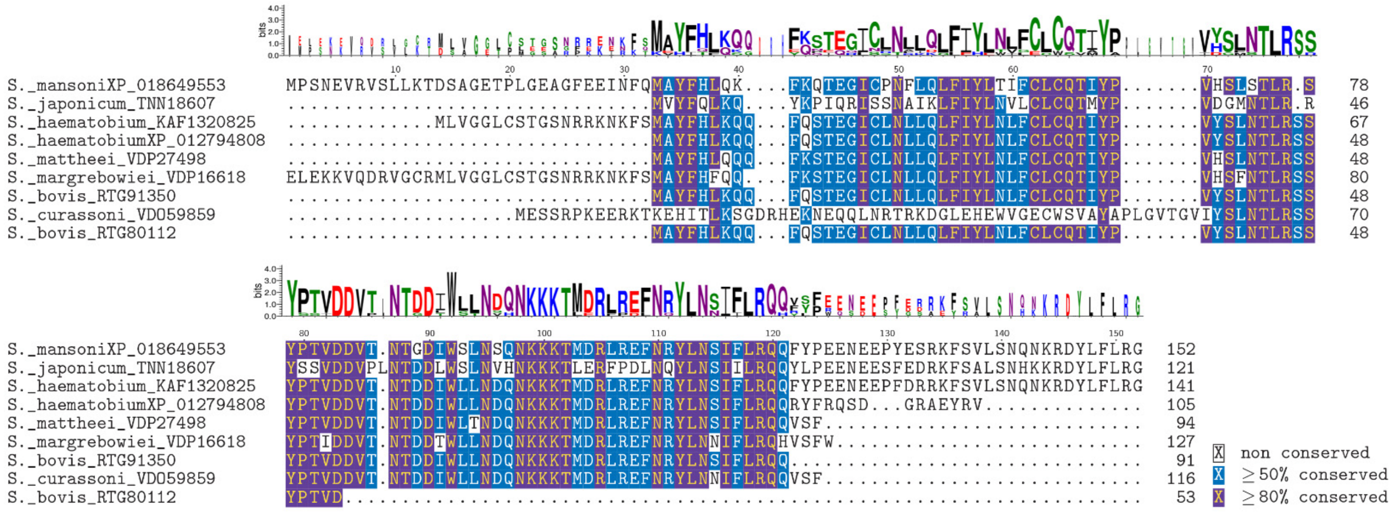
S._mansoniXP_018649553
S._japonicum_TNN18607
S._haematobium_KAF1320825
S._haematobiumXP_012794808
S._mattheei_VDP27498
S._margrebowiei_VDP16618
S._bovis_RTG91350
S._curassoni_VDO59859
S._bovis_RTG80112
non conserved
≥50% conserved
≥80% conserved

Npp-32

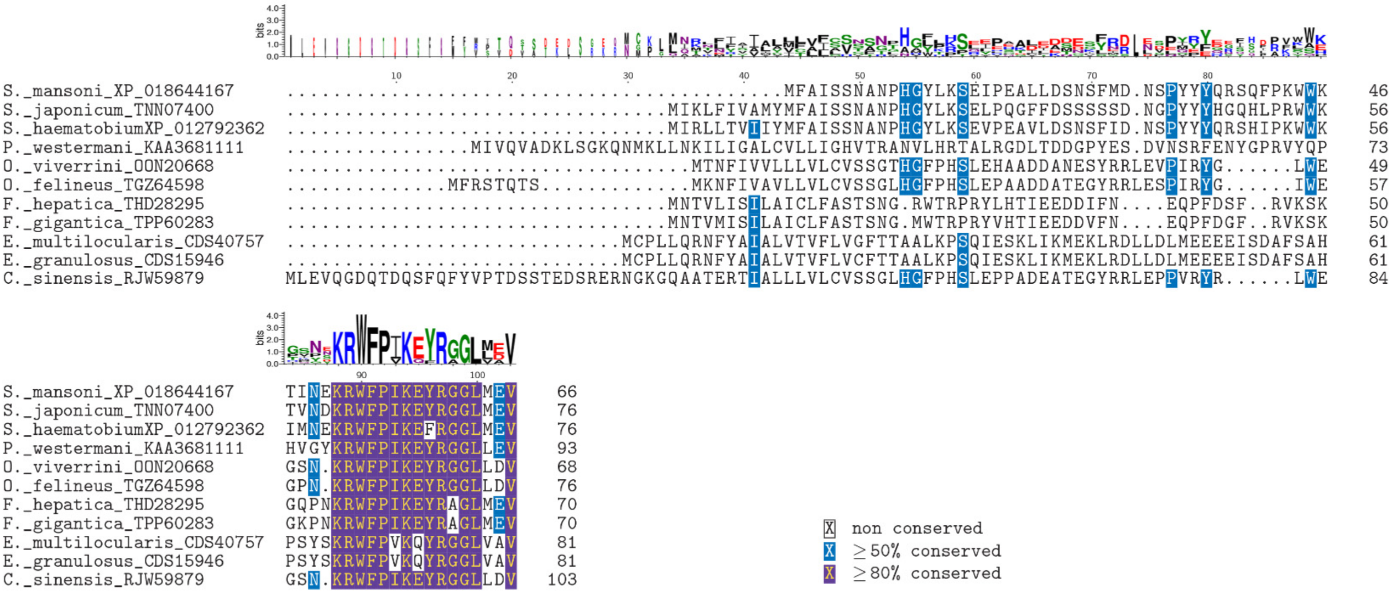

Npp-33

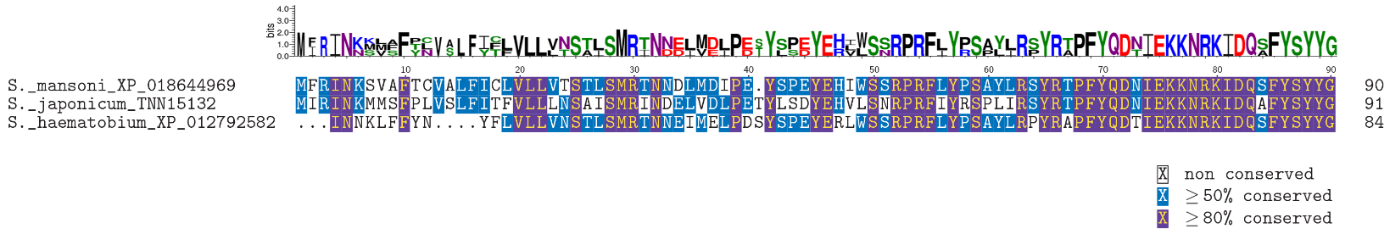

Npp-34

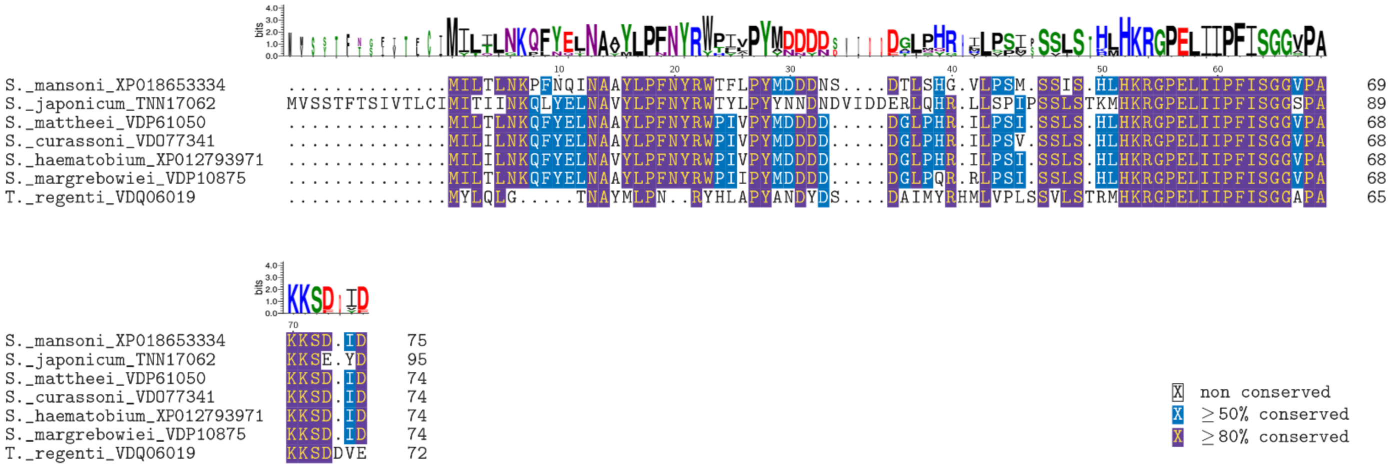

Npp-35

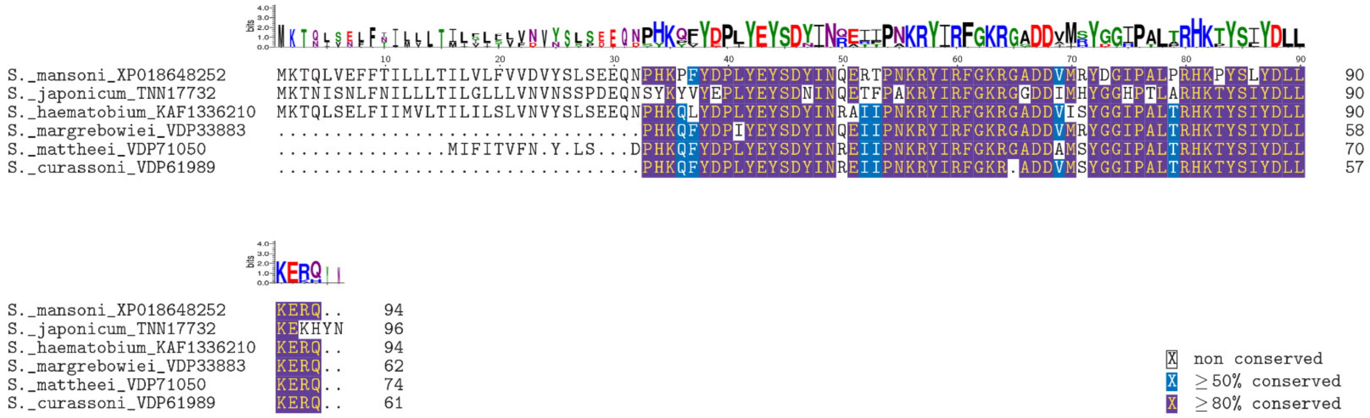

Npp-36

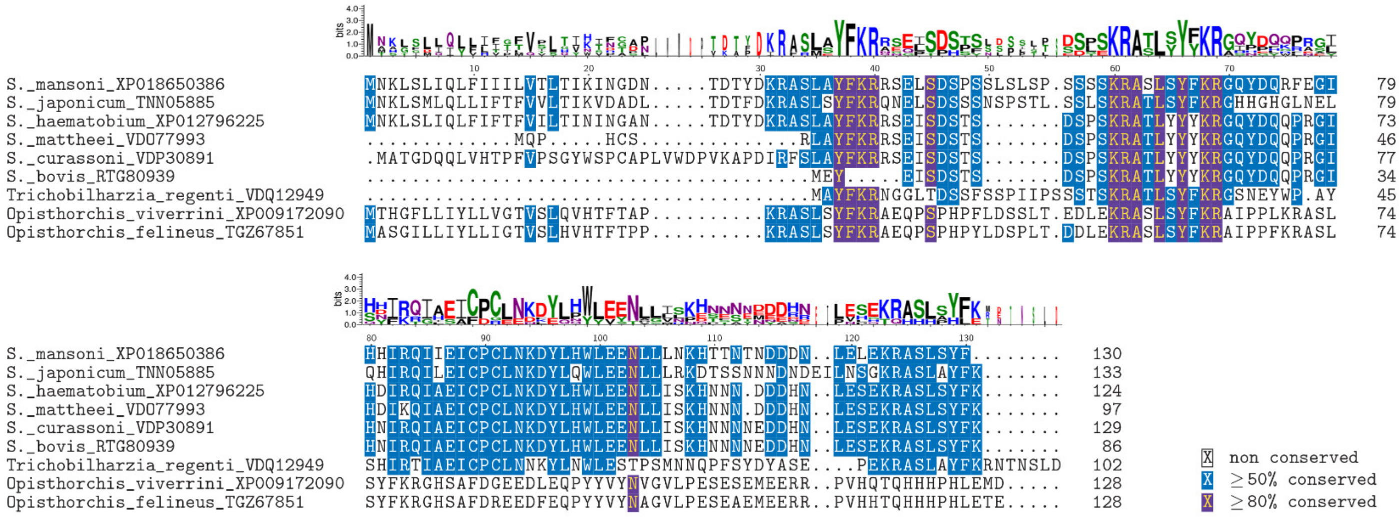

Npp-37

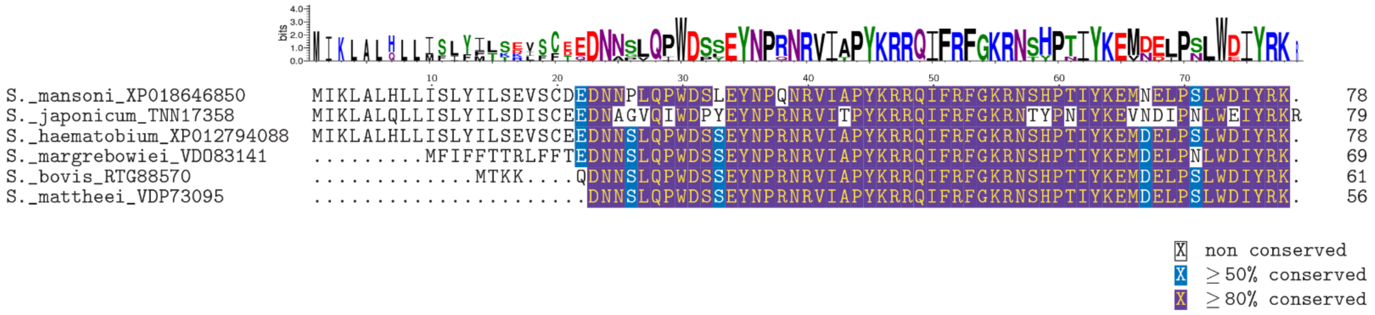

Npp-38

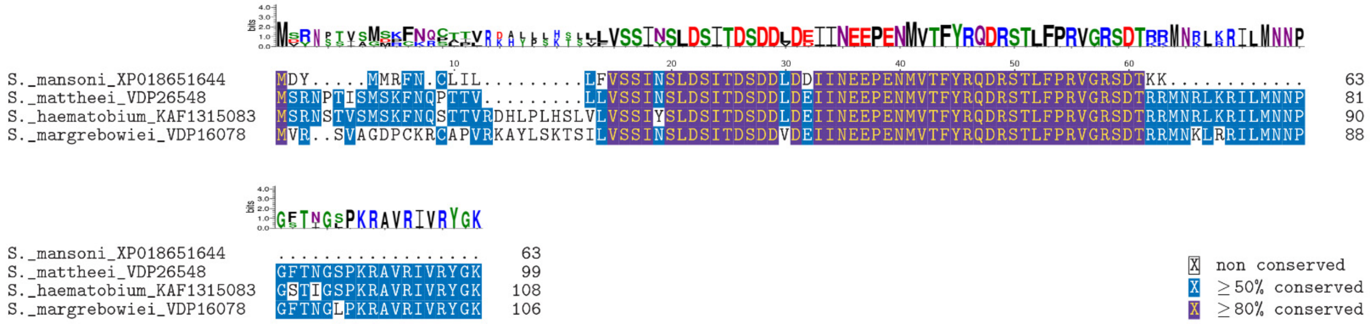
S._mansoni_XP018651644 MDY.....MMRFN.CLIL.........LFVSSINSLDSITDSDDLDDIINEEPENMVTFYRQDRSTLFPRVGRSDTKK............ 63
S._mattheei_VDP26548 MSRNPTISMSKFNQPTTV.........LLVSSINSLDSITDSDDLDEIINEEPENMVTFYRQDRSTLFPRVGRSDTRRMNRLKRILMNNP 81
S._haematobium_KAF1315083 MSRNSTVSMSKFNQSTTVRDHLPLHSLVLVSSIYSLDSITDSDDLDEIINEEPENMVTFYRQDRSTLFPRVGRSDTRRMNRLKRILMNNP 90
S._margrebowiei_VDP16078 MVR..SVAGDPCKRCAPVRKAYLSKTSILVSSINSLDSITDSDDVDEIINEEPENMVTFYRQDRSTLFPRVGRSDTRRMNKLRRILMNNP 88
S._mansoni_XP018651644 63
S._mattheei_VDP26548 GFTNGSPKRAVRIVRYGK 99
S._haematobium_KAF1315083 GSTIGSPKRAVRIVRYGK 108
S._margrebowiei_VDP16078 GFTNGLPKRAVRIVRYGK 106
X non conserved
X ≥50% conserved
X ≥80% conserved

Npp-39

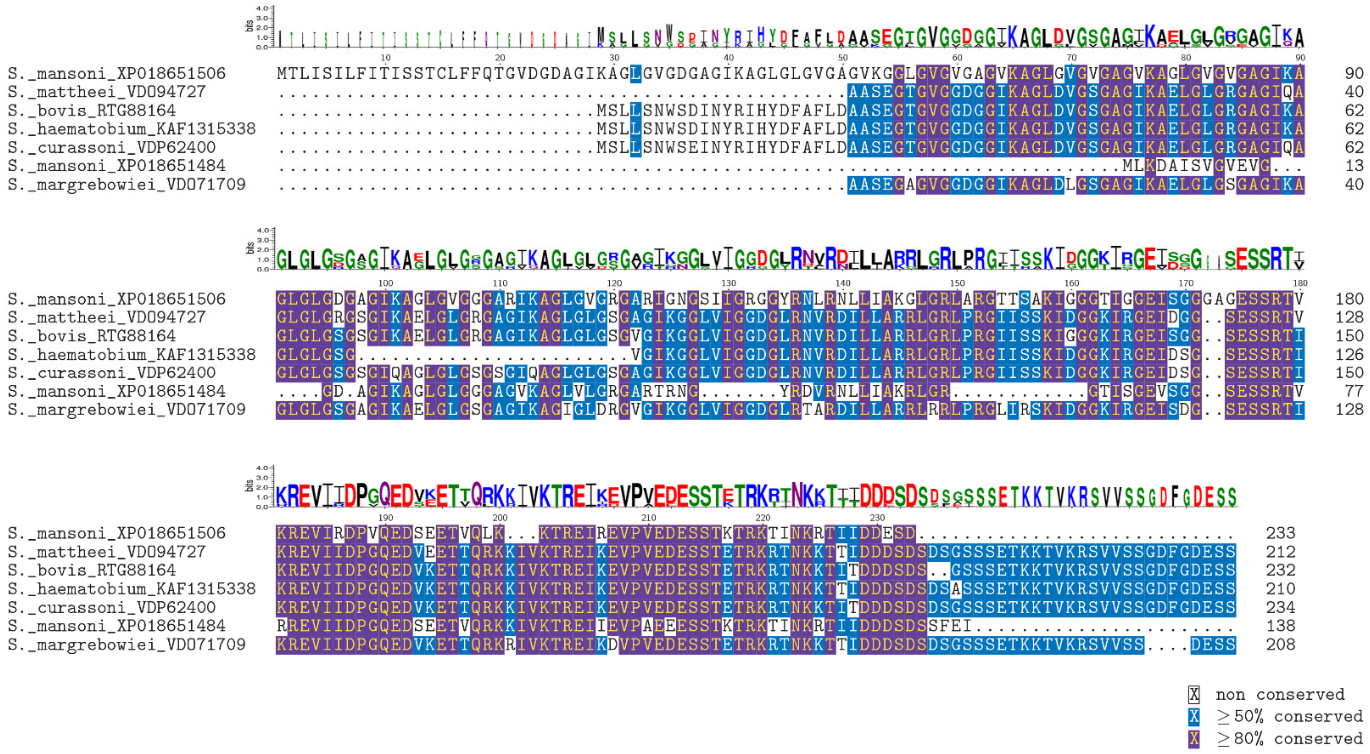
S._mansoni_XP018651506 MTLISILFITISSTCLFFQTGVDGDAGIKAGLGVGDGAGIKAGLGLGVGAGVKGGLGVGVGAGVKAGLGVGVGAGVKAGLGVGVGAGIKA 90
S._mattheei_VD094727 ....AASEGTGVGGDGGIKAGLDVGSGAGIKAELGLGRGAGIQA 40
S._bovis_RTG88164 ............................MSLLSNWSDINYRIHYDFAFLDAASEGTGVGGDGGIKAGLDVGSGAGIKAELGLGRGAGIKA 62
S._haematobium_KAF1315338 ............................MSLLSNWSDINYRIHYDFAFLDAASEGTGVGGDGGIKAGLDVGSGAGIKAELGLGRGAGIKA 62
S._curassoni_VDP62400 ............................MSLLSNWSEINYRIHYDFAFLDAASEGTGVGGDGGIKAGLDVGSGAGIKAELGLGRGAGIQA 62
S._mansoni_XP018651484 ....MLKDAISVGVEVG... 13
S._margrebowiei_VD071709 ....AASEGAGVGGDGGIKAGLDLGSGAGIKAELGLGSGAGIKA 40
S._mansoni_XP018651506 GLGLGDGAGIKAGLGVGGGARIKAGLGVGRGARIGNGSIIGRGGYRNLRNLLIAKGLGRLARGTTSAKIGGGTIGGEISGGGAGESSRTV 180
S._mattheei_VD094727 GLGLGRGSGIKAELGLGRGAGIKAGLGLGSGAGIKGGLVIGGDGLRNVRDILLARRLGRLPRGIISSKIDGGKIRGEIDGG..SESSRTV 128
S._bovis_RTG88164 GLGLGSGSGIKAELGLGRGAGIKAGLGLGSGVGIKGGLVIGGDGLRNVRDILLARRLGRLPRGIISSKIGGGKIRGEISGG..SESSRTI 150
S._haematobium_KAF1315338 GLGLGSG...........................VGIKGGLVIGGDGLRNVRDILLARRLGRLPRGIISSKIDGGKIRGEIDSG..SESSRTI 126
S._curassoni_VDP62400 GLGLGSGSGIQAGLGLGSGSGIQAGLGLGSGAGIKGGLVIGGDGLRNVRDILLARRLGRLPRGIISSKIDGGKIRGEIDSG..SESSRTI 150
S._mansoni_XP018651484 ....GD.AGIKAGLGLGGGAGVKAGLVLGRGARTRNG.......YRDVRNLLIAKRLGR.............GTISGEVSGG..SESSRTV 77
S._margrebowiei_VD071709 GLGLGSGAGIKAELGLGSGAGIKAGIGLDRGVGIKGGLVIGGDGLRTARDILLARRLRRLPRGLIRSKIDGGKIRGEISDG..SESSRTI 128
S._mansoni_XP018651506 KREVIRDPVQEDSEETVQLK...KTREIREVPVEDESSTKTRKTINKRTIIDDESD............................. 233
S._mattheei_VD094727 KREVIIDPGQEDVEETTQRKKIVKTREIKEVPVEDESSTETRKRTNKKTTIDDDSDSDSGSSSETKKTVKRSVVSSGDFGDESS 212
S._bovis_RTG88164 KREVIIDPGQEDVKETTQRKKIVKTREIKEVPVEDESSTETRKRTNKKTITDDDSDS..GSSSETKKTVKRSVVSSGDFGDESS 232
S._haematobium_KAF1315338 KREVIIDPGQEDVKETTQRKKIVKTREIKEVPVEDESSTETRKRTNKKTTIDDDSDSDSASSSETKKTVKRSVVSSGDFGDESS 210
S._curassoni_VDP62400 KREVIIDPGQEDVKETTQRKKIVKTREIKEVPVEDESSTETRKRTNKKTITDDDSDSDSGSSSETKKTVKRSVVSSGDFGDESS 234
S._mansoni_XP018651484 RREVIIDPGQEDSEETVQRKKIVKTREIIEVPAEEESSTKTRKTINKRTIIDDDSDSSFEI....................... 138
S._margrebowiei_VD071709 KREVIIDPGQEDVKETTQRKRIVKTREIKDVPVEDESSTETRKRTNKKTTIDDDSDSDSGSSSETKKTVKRSVVSS....DESS 208
X non conserved
X ≥50% conserved
X ≥80% conserved

Npp-40

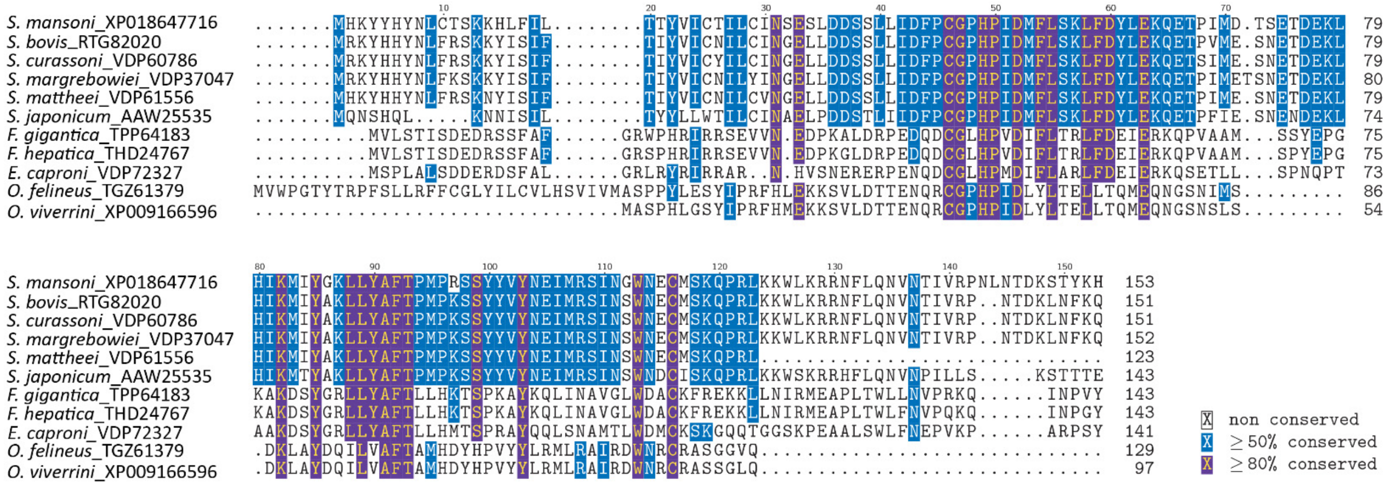

References:

Kumar, S., et al. (2018). "MEGA X: Molecular Evolutionary Genetics Analysis across Computing Platforms." Mol Biol Evol **35**(6): 1547-1549.

The Molecular Evolutionary Genetics Analysis (Mega) software implements many analytical methods and tools for phylogenomics and phylomedicine. Here, we report a transformation of Mega to enable cross-platform use on Microsoft Windows and Linux operating systems. Mega X does not require virtualization or emulation software and provides a uniform user experience across platforms. Mega X has additionally been upgraded to use multiple computing cores for many molecular evolutionary analyses. Mega X is available in two interfaces (graphical and command line) and can be downloaded from www.megasoftware.net free of charge.

Crooks GE, Hon G, Chandonia JM, Brenner SE WebLogo: A sequence logo generator, Genome Research, 14:1188-1190, (2004)
