# Supplementary figures and images for: Identification of Putative Neuropeptides That Alter the Behaviour of Schistosoma mansoni Cercariae
Source: Biology (Basel). 2022 Sep 12;11(9):1344. doi: 10.3390/biology11091344 (PMC9495596; doi:10.3390/biology11091344)

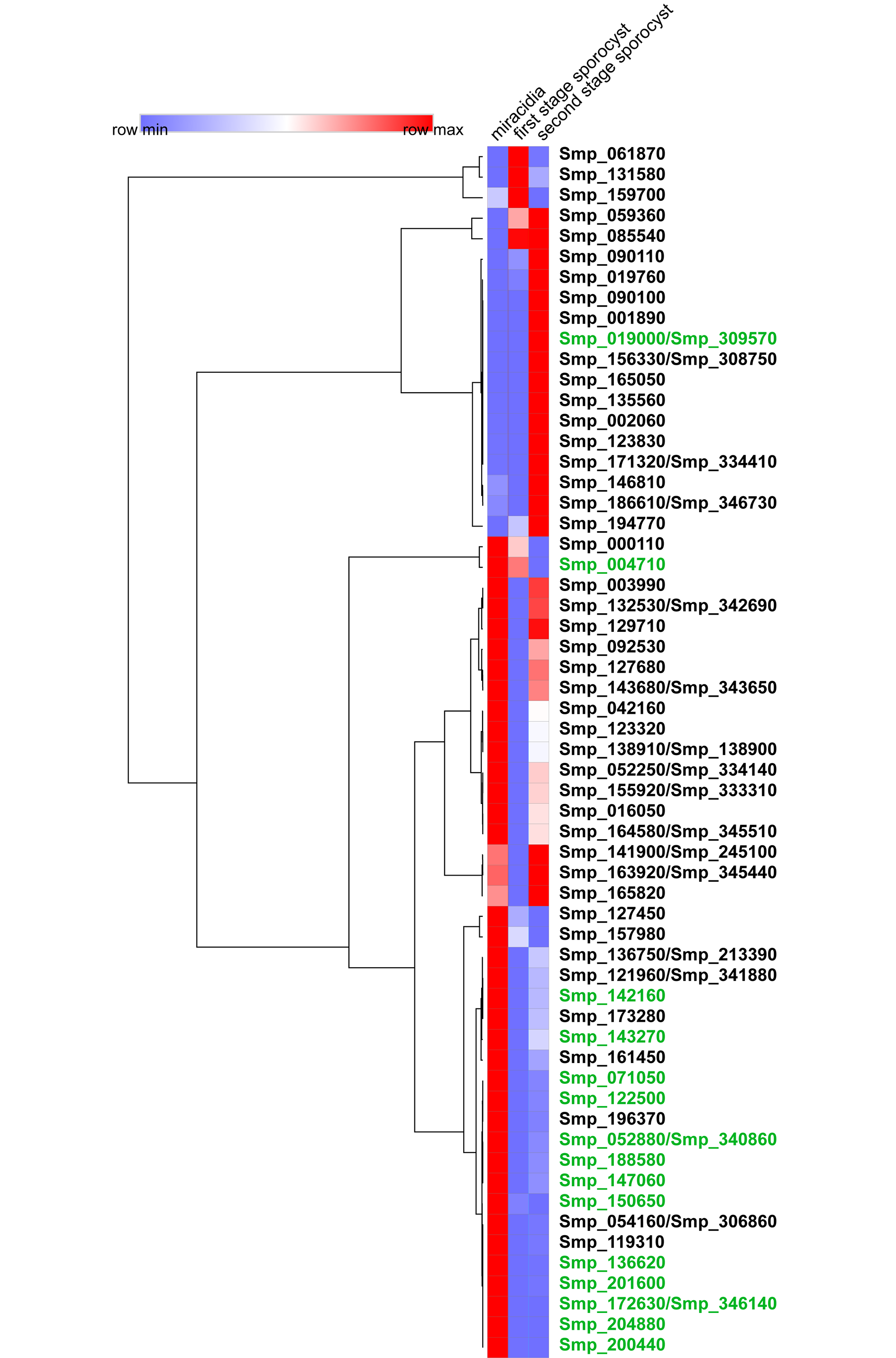

Supplement: Supplementary file 1 [file biology-11-01344-s001.zip › Figure S1.tif]

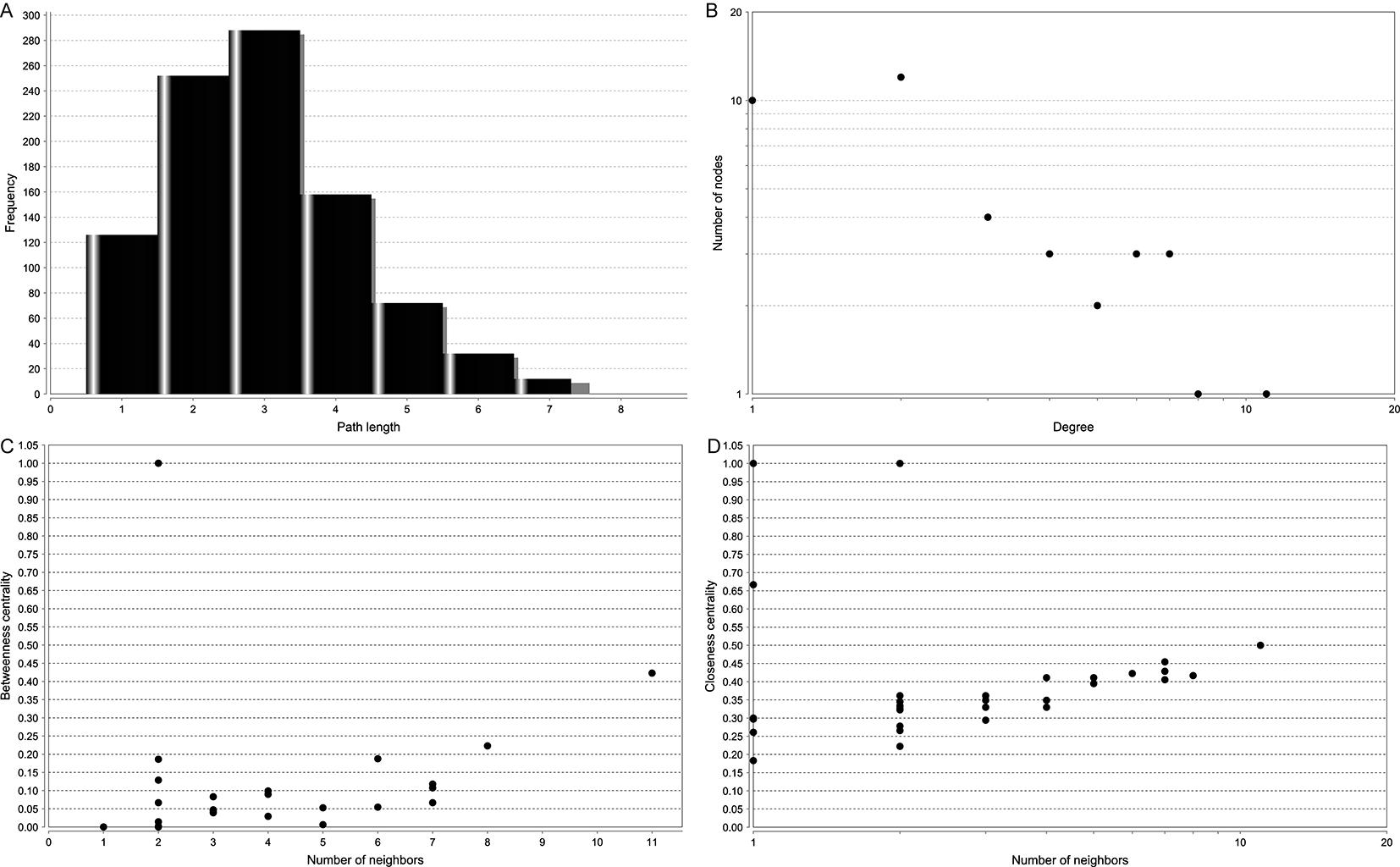

Supplement: Supplementary file 1 [file biology-11-01344-s001.zip › Figure S2.tif]

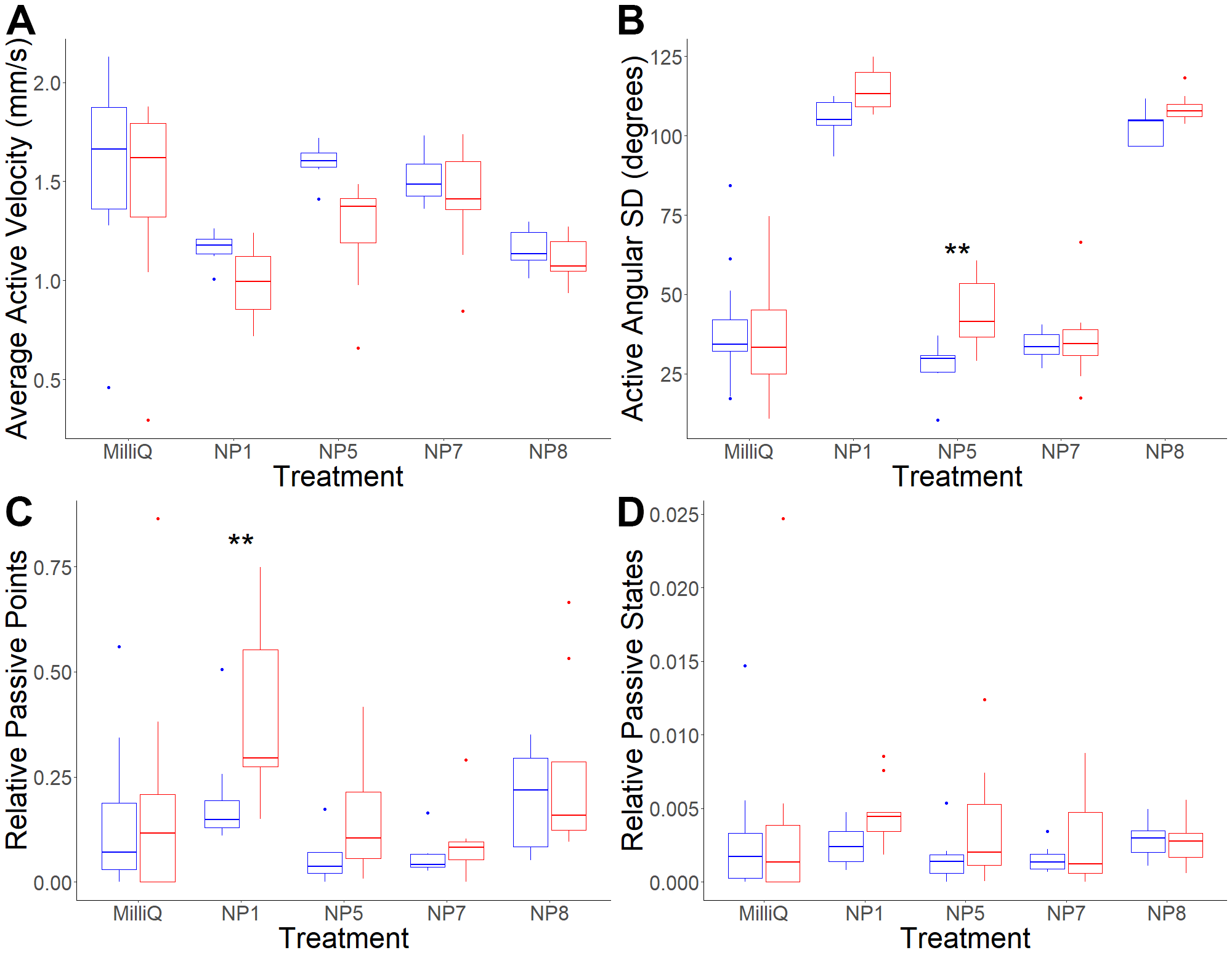

Supplement: Supplementary file 1 [file biology-11-01344-s001.zip › Figure S3.tif]

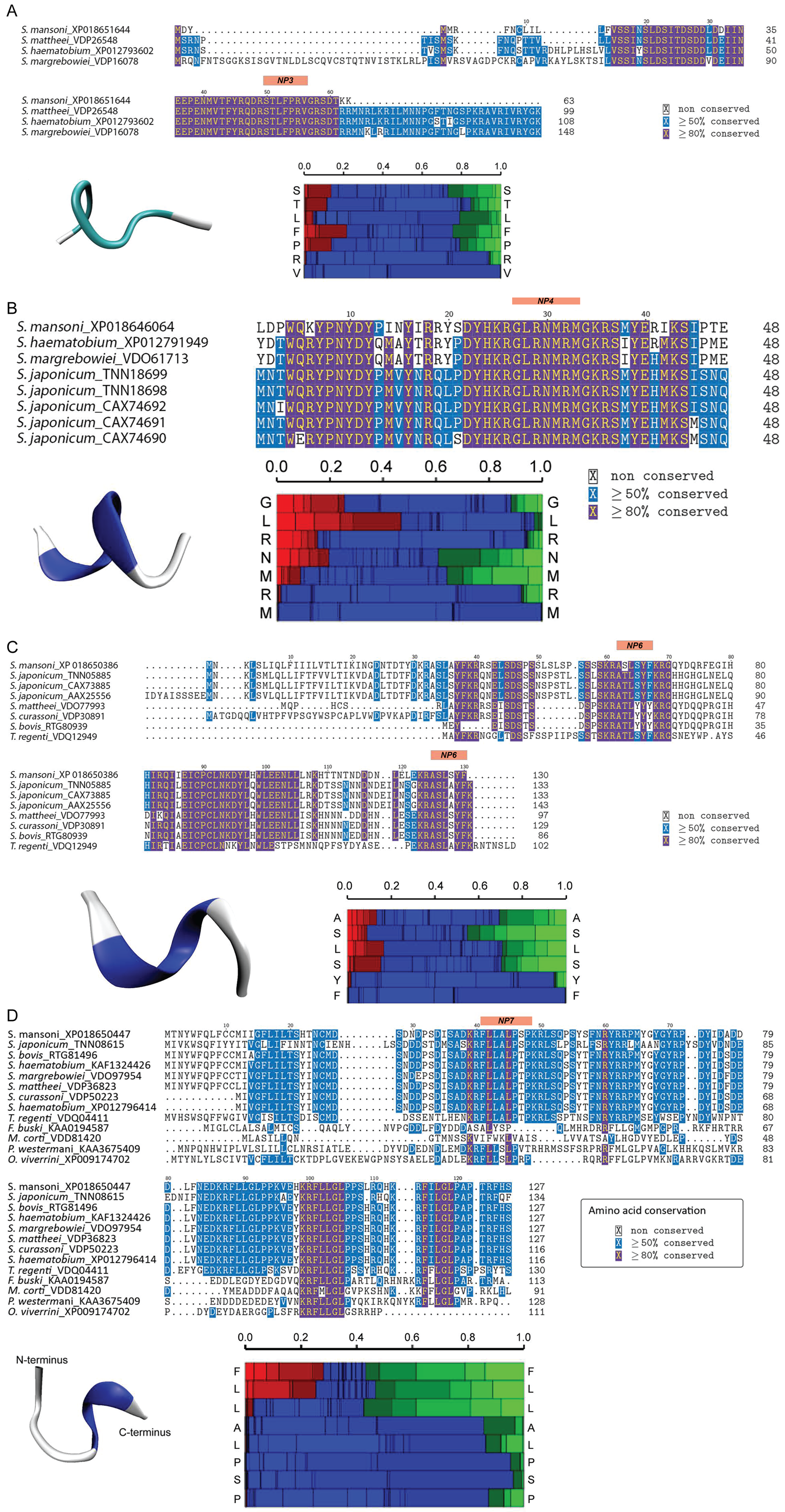

Supplement: Supplementary file 1 [file biology-11-01344-s001.zip › Figure S4.tif]
